# A closed audit reviewing the electrocardiograms of patients presenting to the memory assessment team

**DOI:** 10.1192/bjo.2021.133

**Published:** 2021-06-18

**Authors:** Joseph Heath, Maroulla Anderson, Jonathan Miles-Stokes

**Affiliations:** Greater Manchester Mental Health NHS Foundation Trust

## Abstract

**Aims:**

To review the ECGs of all patients referred to MAT services over the preceding 5 year period.

**Background:**

Neurodegenerative conditions such as Alzheimer's Disease can be treated with Acetylcholinesterase Inhibitors (AChEI) to slow down cognitive decline. Side effects of AChEIs include bradycardia, syncope and cardiac conduction disorders. An electrocardiograms (ECG) is completed prior to memory assessment team (MAT) medical assessments to screen for those who may be at risk of the cardiac side effects of AChEIs. ECGs may be included in the initial referral to the service or completed by the MAT. Given the predominantly elderly population referred to the MATs service, other incidental abnormalities are to be expected. Not all MAT referrals that are screened by memory nurses reach the threshold to be reviewed by the medical team and therefore not all ECGs are routinely reviewed, potentially missing clinically significant abnormalities.

**Result:**

A total of 1795 patients were identified as being referred to a single mental health unit in the North West on England over a five-year period. 781 (44%) of the patients had an ECG completed by the MAT, of which 452 (58%) showed an abnormality. Significant abnormalities that were previously unknown to the patients’ primary care provider include eight cases of Atrial Fibrillation (AF), four cases of Trifasciular Block, and 19 cases of Left Ventricular Hypertrophy (LVH). 64 (8%) of patients who had an ECG by the MAT had a bradycardia.

**Conclusion:**

In addition to identifying abnormalities that could interfere with memory medication, this audit showed that over half of the ECGs completed by the MAT had an atypical trace. Cardiology was consulted to identify which abnormalities were considered clinically significant and if not already known, the general practitioner (GP) was informed. A change in the local service means that all ECGs completed by the MAT are now screened at point of filling into the notes, so any future abnormalities are identified and followed up immediately.

